# Changing Trends in Oesophageal Endoscopy: A Systematic Review of Transnasal Oesophagoscopy

**DOI:** 10.1155/2013/586973

**Published:** 2013-08-01

**Authors:** Junainah Sabirin, Maharita Abd Rahman, Philip Rajan

**Affiliations:** ^1^Health Technology Assessment Section, Ministry of Health Malaysia, Federal Government Administrative Centre, 62590 Putrajaya, Malaysia; ^2^Hospital Raja Permaisuri Bainun and University Sains Malaysia and University Malaya and Jalan Hospital, 30990 Ipoh, Perak, Malaysia

## Abstract

The safety, efficacy, and economic implications of using transnasal oesophagoscopy (TNE) are compared with conventional rigid or flexible oesophagoscopy for oesophageal disorders in otorhinolaryngology (ORL) clinics in this systematic review. Eleven electronic databases were searched for articles on transnasal oesophagoscopy. A total of 67 relevant titles were identified and 39 abstracts were screened of which 17 full- text articles were included in this report. There was fair level of evidence to suggest that TNE was effective for screening examination in patients with dysphagia, globus pharyngeus, and reflux symptoms and for detection of metachronous oesophageal carcinoma. TNE can also be used to biopsy suspicious lesions in the upper aerodigestive tract, placement of wireless pH capsule, transnasal balloon dilation of the oesophagus, secondary tracheoesophageal puncture, and management of foreign bodies. TNE was well tolerated and can be safely performed in an office setting with topical anaesthesia. Complications associated with TNE were mild and uncommon. There was evidence to suggest potential cost savings by performing TNE in the office setting compared with conventional investigation and examination for dysphagia. TNE may lead to a change in practice from investigation and treatment in the operating theatre or day care center to an office-based practice.

## 1. Introduction

Oesophagoscopy and barium radiology represents the primary means by which structural diseases of the oesophagus are investigated. Until 1996, the oesophagoscopy performed by otolaryngologists had primarily been rigid endoscopy, performed transorally, with patients under general anaesthesia. Beginning mid 1990s, otolaryngologists began to perform oesophagoscopy utilizing an ultrathin, flexible scopes passed transnasally, with the patients not sedated, solely relying on topical anaesthesia. This approach is called transnasal oesophagoscopy (TNE) in the United States of America (USA), and it is known as transnasal flexible laryngooesophagoscopy (TNFLO) in the United Kingdom (UK). TNE is performed in the clinic without the sophisticated patient monitoring and skilled ancillary personnel that are required during and after rigid oesophagoscopy. It is performed with conscious sedation in the endoscopy suite or room. It is claimed to have the following advantages over conventional peroral, rigid, or flexible oesophagoscopy carried out in a sedated patient: enhanced patient safety, improved survival of oesophageal adenocarcinoma, increased practice efficiency, and costsaving [1].

 Indications for TNE include dysphagia, oesophageal symptoms that persist despite an appropriate trial of therapy, odynophagia, screening, and possibly surveillance for Barrett's oesophagus, caustic ingestion evaluation, foreign body evaluation and possible removal, globus pharyngeus, chronic cough, cervical dysphagia, head and neck cancer, poorly controlled asthma, and moderate to severe laryngopharyngeal reflux (LPR) [2, 3]. TNE may be used to perform a wide variety of procedures. These include biopsies, use of lasers, oesophageal dilation, secondary tracheoesophageal puncture, treatment of achalasia, feeding tube insertion, and the insertion of wireless pH capsule [2–7]. 

The transnasal oesophagoscope ranges in diameter from 4.5 mm to 5.1 mm. There are two different types of TNE systems available [8]. One is a video chip flexible endoscope system where the camera is located at the distal tip of the endoscope and the scope is attached to a video processor. The other is an add-on camera flexible endoscope system in which a camera is attached to the proximal portion of the fiberscope, usually at the eyepiece. The fiber optic add-on camera system can incorporate a single-use, disposable TNE EndoSheath. The distal chip endoscopes and endosheaths have a channel for air insufflation or water instillation and for suction. A working channel allowing passage of 1.8 mm cup forceps, biopsy brushes, or flexible lasers is also available [1, 8]. Transnasal esophagoscope has United States of America Food and Drug Regulation (USFDA) approval [9].

Transnasal oesophagoscopy is an office-based procedure. The patient is seated in a standard ENT examining chair. No cardiac monitoring is necessary unlike the conscious sedation. Although not absolutely necessary, it is preferable that the patient does not eat or drink for at least three hours before TNE. This ensures the stomach is empty during the examination. No conscious or intravenous sedation is used. The key to successful examination is adequate topical nasal anaesthesia and decongestion [3, 8].

This systematic review was conducted to look into the safety, efficacy, or effectiveness and economic implications of TNE and to compare it with the conventional peroral, rigid, or conventional flexible esophagoscopy (CE). 

## 2. Methodology 

Eleven electronic databases were searched for articles on transnasal oesophagoscopy (TNE). The following databases were searched through the Ovid interface: MEDLINE(R) In-process and other Non-Indexed Citations and Ovid MEDLINE(R) 1948 to present, EBM Reviews—Cochrane Database of Systematic Reviews (2005 to June 2011), EBM Reviews—Cochrane Central Register of Controlled Trials (3rd Quarter 2011), EBM Reviews—Database of Abstracts of Review of Effects (3rd Quarter 2011), EBM Reviews—Health Technology Assessment (3rd Quarter 2011), EBM Reviews—NHS Economic Evaluation Database (3rd Quarter 2011). Parallel searches were run in PubMed. No limits were applied to the search. No filters were applied. Other databases searched include INAHTA database, Horizon Scanning databases (Australia and New Zealand Horizon Scanning, Defra-UK Horizon Scanning, and National Horizon Scanning Centre), ASERNIP-S, and FDA database. The last search was run on July 15, 2011. Additional articles were identified from reviewing the bibliographies of retrieved articles and contacting the authors. All relevant articles comprising randomized controlled trials, cross-sectional studies, reviews, and case reports were included. All the relevant literature was appraised using the Critical Appraisal Skills Programme (CASP) and evidence was graded based on guidelines from US/Canadian Preventive Services Task Force and NHS Centre for Reviews and Dissemination (CRD) University of York, Report Number 4 (2nd Edition), March 2001 for test accuracy studies [10, 11]. Full details on the review committee, methodology, evidence, and findings can be obtained from the HTA report at http://www.moh.gov.my/v/hta or from the authors.

## 3. Results

**Figure 1:**
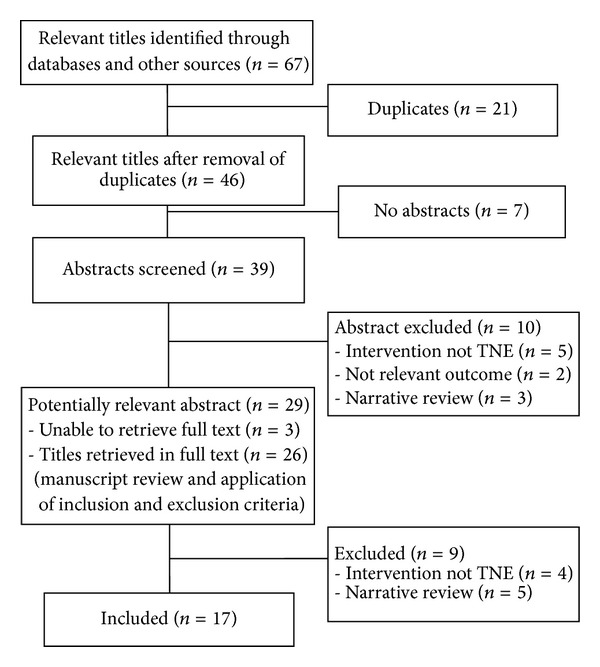
Flow chart of study selection.

A total of 67 relevant titles were identified and 39 abstracts were screened using the inclusion and exclusion criteria and 17 full text articles were included in this report. The articles comprised of one cross-sectional diagnostic study, 13 cross-sectional studies, two cross-sectional studies with economic evaluation, and one case report. The search did not yield any health technology assessment reports, systematic reviews, or randomized controlled trials related to TNE (Figure 1).

## 4. Efficacy or Effectiveness of TNE

### 4.1. Detection of Oesophageal and Extraoesophageal Lesions 

#### 4.1.1. Screening Examination in Patients with Dysphagia or Globus Pharyngeus or Reflux Symptoms

Since the introduction of TNE, there have been several studies conducted by otolaryngologists in the USA, UK, Japan, and Taiwan. The first article by an otolaryngologist on TNE was published in 2001 by Aviv et al. who conducted a cross-sectional study in Columbia University, USA using TNE in an office setting among 14 patients with dysphagia. There was no significant difference between the larynx and oesophagus in terms of quality of optical image; larynx (mean, 1.1; range, 1 to 2, standard deviation (SD), 0.1) and oesophagus (mean, 2.1; range, 1 to 6, SD, 0.3) whereby 1 represented excellent image quality and 10 represented very poor image quality. They found that all patients completed the TNE with the oesophagus readily intubated and oesophageal mucosa clearly visualized. Pathological oesophageal findings included a proximal oesophageal stricture, a patulous upper oesophageal sphincter, and a Zenker's diverticulum. Incidental finding of left nasopharyngeal mass was noted in one patient [12]. 

The largest consecutive report of TNE examinations has been published by Postma et al. in USA in which they reported on 700 consecutive patients. A total of 611 consecutive patients who underwent TNE using VE-1530, Pentax Precision Instrument Corporation, Orangeburg, New York, between January 28, 2001 and January 1, 2004 were compared with 100 consecutive patients previously reported. The patients presented primarily with reflux or globus pharyngeus and/or dysphagia. Only 2.8% of procedures (17) were aborted secondary to an inability to pass the endoscope through a tight nasal vault and 0.3% (two) were aborted secondary to self-limited vasovagal responses. Significant findings were noted in 49.7% (294/592) of patients. The most common findings in the oesophagus were oesophagitis (17.0%), hiatal hernia (8.0%), Barrett's metaplasia (5.0%), candidiasis (5.0%), stricture (4.0%), and carcinoma (4.0%) [13]. The results were similar to their initial report of 100 consecutive patients whereby significant findings were found in 43.7% (42/96) of patients and four procedures (4.0%) were aborted secondary to a tight nasal vault [14]. The authors concluded that TNE may replace radiographic imaging of the oesophagus in otolaryngology patients with reflux, globus pharyngeus, and dysphagia [13, 14].

 Andrus et al. who evaluated the findings of TNE conducted among 30 patients presenting with dysphagia, GER, or LPR and oesophageal surveillance for head and neck cancer found that 43.3% of them had positive findings and patient management was affected after TNE. Findings include Barrett's oesophagus, candidal oesophagitis, posterior glottis oedema, postcricoid mass, oesophageal diverticulum, oesophageal dysmotility, oesophageal stricture, patulous oesophagus, and gastritis. Patients with Barrett's oesophagus were referred to a gastroenterologist for further evaluation and management. A patient with oesophageal stricture was dilated successfully with a number 36 bougie dilator at the time of TNE and being followed symptomatically. Patients with negative examinations were followed by the otolaryngologist. Seven patients who would normally have been evaluated with a barium swallow for globus pharyngeus or dysphagia did not undergo the test as a result of negative TNE [15].

Similarly, Price et al. demonstrated the diagnostic capabilities of TNFLO using Pentax 80 K series digital video endoscope (EE 1580 K, Pentax, Slough, UK) in the UK. A total of 116 TNFLO procedures were performed. Indications for TNFLO include screening examination for symptoms of globus pharyngeus, dysphagia, dysphonia, and head and neck cancer. All patients were investigated and treated under local anaesthesia and no patients required sedation or any other medication. Only 1.8% (1/56) of patients with globus pharyngeus did not tolerate the procedure. Of the 56 patients with globus pharyngeus, 10.7% (6/56) of patients were found to have pathology and 87.5% (49/56) of patients with no identifiable pathology were discharged. Among patients with dysphagia, lesions such as foreign bodies, complete oesophageal stenosis, benign oesophageal stricture, postcricoid tumour, postcricoid webs, and benign pharyngeal pouches were identified. As for 14 patients who presented with dysphonia the findings were that one had normal larynx, one had Reinke's oedema, one had vocal cord palsy (had vocal cord medialization procedure using TNFLO), ten had suspicious lesions of the larynx and biopsies were taken, and one had recurrent respiratory papillomatosis which was successfully treated with Nd-YAG laser [16]. 

Another cross-sectional study conducted by McPartlin et al. among 16 patients with symptoms of globus pharyngeus or mild dysphagia at Cross Hospital, London, UK, found that 43.7% of patients had mild Gastroesophageal reflux (GORD) or GORD. Quality of views obtained were rated as excellent in 62.5% (10/16), good in 31.3% (5/16), and fair in 6.2% (1/16). None were rated as poor [17].

The utility of office-based TNE in the evaluation of patients with chronic dysphagia, globus sensation, and a sensation of “food sticking” with swallowing was also demonstrated by Kumar and Amir in their two case reports at the University College of Medicine, Philadelphia, Pennsylvania, U.S.A. In the first case, TNE revealed a solitary midoesophageal diverticulum with otherwise normal mucosa. The patient underwent endoscopic diverticulectomy. On follow-up office visits, the patient reported resolution of his dysphagia symptoms and resumption of a regular diet. In the second case, TNE revealed multiple diverticula and a tortuous oesophagus. The patient underwent endoscopic staple-assisted diverticulectomy of the Zenker's diverticulum and also underwent esophagoscopy and dilation. The patient's swallowing impairments improved and he was subsequently able to tolerate solid foods [18]. 

 In contrast, Koufman et al. from Wake Forest University, U.S.A., in their cross-sectional study involving 58 patients with pH documented LPR, who underwent TNE with biopsies, found that the prevalence of oesophagitis and Barrett's metaplasia was 19.0%. They concluded that these data confirm the clinical impression that the patterns, mechanisms, and manifestations of LPR differ from those of classic oesophageal reflux disease. Unlike GERD, patients with LPR uncommonly have oesophagitis. Thus, although oesophagoscopy may be an excellent method for screening the oesophagus, it is not the method of choice for diagnosing LPR [19]. 

#### 4.1.2. Patients with Head and Neck Cancer

Panendoscopy is part of the standard evaluation of individual with head and neck squamous cell carcinoma. Often, these patients possess comorbidities that increase the risk of general anaesthesia. In-office TNE allows an examination of the aerodigestive tract without the morbidity of anaesthesia. TNE has been demonstrated to be safe and well tolerated in the vast majority of patients including those with head and neck cancer [13, 14, 16]. The role of TNE in head and neck oncology was further emphasized by Postma et al. [20]. Transnasal oesophagoscopy was performed in a prospective fashion in 17 patients with known lesions of the upper aerodigestive tract in Wake Forest University, U.S.A. All masses were suspected to be malignant. Each of these individuals underwent TNE with biopsies and soon thereafter went to the operating room for standard panendoscopy with biopsies. Their findings were entirely congruent between the two modalities. TNE provided 100% sensitivity and specificity in biopsy results and staging of the tumour when compared with standard panendoscopy. The authors concluded that TNE gives the surgeon the ability to perform a remarkable variety of procedures in the outpatient setting without sedation. It may obviate the need for routine panendoscopy in head and neck cancer patients [20].

The ability of TNE for detecting metachronous oesophageal squamous carcinoma in patients with head and neck squamous cell carcinoma (HNSCC) was evaluated by Su et al. at a tertiary medical centre in Taiwan [21]. In total, TNE was performed 398 times in 293 previously treated patients with HNSCC between December 2007 and January 2009. The site distributions of HNSCC were 63% in the oral cavity, 14% in the oropharynx, 15% in hypopharynx, and 8% in the larynx. Metachronous oesophageal squamous carcinoma was detected in 5.1% (15/293) of patients. The median time to the diagnosis of oesophageal carcinoma was 15 months (range, 7–76 months). Eleven (73.0%) of them were found within three years after HNSCC was diagnosed. The prevalence rate was 15.9% (7/44) in patients with hypopharyngeal cancer which is significantly higher than the 8.3% (2/24) in laryngeal, 7.1% (3.42) in oropharyngeal, and 1.6% (3/183) in oral cancer (*P* = 0.001). The stage distributions of oesophageal squamous carcinoma were I-II in 80% (12/15) and III-IV in 20% (3/15) of patients. Subsequently, curative strategies were performed in 87% (13/15) of patients [21]. 

### 4.2. TNE Assisted Procedures 

#### 4.2.1. Biopsy of Suspicious Lesions in the Upper Aerodigestive Tract

Studies have reported the use of TNE for biopsies of suspicious lesions in the upper aerodigestive tract. Postma et al. and Belafsky et al. described the use of TNE for biopsy of suspicious lesions in the laryngopharynx, while Price et al. described the use of TNE for biopsy of suspicious lesions in the larynx, postnasal space, and the uvula [13, 14, 16]. In a study by Belafsky et al., five of the eight biopsies (63%) resulted in a diagnosis of squamous cell carcinoma; one biopsy found a laryngeal fungal infection and two were nondiagnostics [14]. Price et al. found that eight of the twelve biopsies in the larynx (66.7%), one of the five biopsies in the postnasal space (20.0%), and one biopsy in the uvula (100.0%) resulted in a diagnosis of invasive squamous cell carcinoma [16]. 

#### 4.2.2. Placement of Wireless PH Capsule

Belafsky et al. prospectively evaluated 46 patients undergoing unsedated TNE and wireless pH capsule placement at the Scripps Centre for Voice and Swallowing La Jolla, California, U.S.A., between January 1, 2003 and July 31, 2003. The indications of the procedure were chronic cough, 39.1% (18/46), GERD, 39.1% (18/46), and LPR, 21.8% (10/46). Of the procedures performed, 85% (39/46) were successful. Of the seven procedures that failed, two capsules could not be passed because of a tight nasal vault, three capsules failed because of technical reasons (early detachment in two and delivery system failure in one), one patient went into laryngospasm and could not complete the capsule placement, and one patient lost a wireless data recorder after a successful capsule placement. They concluded that transnasal placement of a wireless pH capsule is a safe and effective diagnostic adjunct to unsedated transnasal oesophagoscopy [22].

Belafsky et al. conducted another study with the aim of evaluating the effect of sedation on the 48-hour wireless pH testing by comparing unsedated pH capsule placement (via transnasal during unsedated TNE, transnasal during unsedated oesophageal manometry, transoral unsedated based on the location of SCJ at a previous EGD) with sedated pH capsule placement (peroral during sedated EGD). They found that the overall reproducibility of the daily pH recordings (day one versus day two) was 77%. All the reflux parameters such as mean reflux episodes, mean time (%) pH < 4, and mean composite score were slightly lower for the sedated group, but the difference was not significant (*P* > 0.05). The authors concluded that intravenous sedation does not appear to have a significant effect on the results of 48-hour wireless pH testing [23].

#### 4.2.3. Transnasal Balloon Dilation of the Oesophagus

The use of TNE in conjunction with balloon dilation of the oesophagus allows the physician an opportunity to dilate all areas of the oesophagus through the nasal cavity. The safety and efficacy of transnasal balloon dilation of the oesophagus was evaluated by Rees CJ, Fordham T, and Belafsky PC at University of California-Davis School of Medicine (UCD) and Wake Forest University School of Medicine (WFU), U.S.A. The cross-sectional study involved a retrospective chart review of all persons undergoing transnasal balloon dilation of the oesophagus at the two universities from January 1, 2007 to December 31, 2008. The study was conducted using Pentax VE-1530 transnasal oesophagoscope (Pentax Precision Medical Co, KayPentax, Lincoln Park, NJ, USA) and multidiameter hydrostatic wire-guided controlled radial expansion oesophageal dilators (Boston Scientific, Natick, MA, USA) [24].

Transnasal balloon dilation of the oesophagus was performed with topical anaesthesia or with the patient under conscious sedation, at the preference of the patients. The study involved 38 patients who presented with cricopharyngeal dysfunction, benign stricture, oesophageal web, and Schatzki ring. Fifty-four transnasal oesophageal balloon dilations were performed in 38 patients. Twenty procedures (37.0%) were performed using topical anaesthesia in the office setting and 34 procedures (63.0%) were performed with conscious sedation in an outpatient surgical suite.The most site of dilation (63.0%) was the upper oesophageal sphincter (UES). Midoesophageal locations were the next most common (26.0%) followed by the LES (7%) and both the EUS and LES (4.0%). Ninety-six point three percent of the procedure was well tolerated. Two procedures (3.7%) were aborted secondary to self-limited laryngospasm or gagging. The authors concluded that transnasal oesophagoscopy balloon dilation can be performed in unsedated or sedated patients with low complications rate. This technique, formerly available only through larger calibre oral gastroscopes and under sedation, allows for office-based oesophageal balloon dilation in an otolaryngology practice [24].

#### 4.2.4. Secondary Tracheoesophageal Puncture (TEP)

Tracheoesophageal puncture (TEP) is a means of restoring voice in patients after laryngectomy. LeBert et al. evaluated the outcomes of voice restoration using office-based TNE to guide the placement of the secondary TEP in three tertiary care medical centres in U.S.A. They conducted a retrospective chart review of patients who underwent TNE-assisted TEP between January 2004 and December 2008. A total of 39 patients were included in the study. Total laryngectomy was the most common surgical procedure underwent by the patients (64.1%, *n* = 25) followed by total laryngectomy with partial pharyngectomy (20.5%, *n* = 8), total laryngopharyngectomy (12.8%, *n* = 5), and one unknown. Eighteen of the 39 patients (46.1%) underwent radiation therapy before surgical treatment [25]. 

They reported an overall success rate of TNE-assisted TEP as 97.4% (38/39) with one unsuccessful attempt (2.6%). There was no statistically significant correlation between patients having undergone radiation therapy or cricopharyngeal myotomy and a successful TEP placement, difficulty in placing the TEP, complications associated with TEP, using the TEP prosthesis, and speech intelligibility at the last follow-up visit (*P* > 0.05). Thirty-one of the thirty-nine patients (79.5%) were still using their TEP prosthesis for speech at the last follow-up visit and 64.5% (20/31) were rated as understandable all the time (PSS-HN Understandability of Speech Subscale Score = 100). They concluded that in-office TNE-assisted TEP placement can safely be performed, with excellent speech outcomes [25].

#### 4.2.5. Management of Foreign Bodies

The utility of TNE for evaluation of possible foreign bodies was reported by Postma et al. and Belafsky et al. [13, 14]. Postma et al. reported that 12 patients underwent TNE for the evaluation of a suspected foreign body. In six patients, a foreign body was found and in five patients the foreign body was pushed during oesophagoscopy into the stomach without difficulty [13]. Belafsky et al. reported that two patients underwent TNE for evaluation of a suspected foreign body whereby both examinations were found to be negative. These patients eventually underwent rigid oesophagoscopy in the operating room under general anaesthesia and were found to be negative [14].

Bennett et al. reported the use of TNE under local anaesthesia for the diagnosis and removal of foreign bodies from the pharynx and oesophagus in adults at the Norfolk and Norwich University Hospitals, UK Five patients aged between 22 and 72 years were examined and found to have foreign bodies involving cod bones (*n* = 2), haddock bone (*n* = 1), plum stone (*n* = 1), and lamb bone (*n* = 1). Two of the foreign bodies were located at the upper oesophagus, one at the pyriform fossa, one at the post cricoids, and one at the base of the tongue. Two of the foreign bodies were extracted via the nose, one extracted via the mouth, one pushed into the stomach, and one removed by direct pharyngoscopy under general anaesthesia. The authors concluded that TNE represents an improvement in the diagnosis and subsequent treatment of a selected group of foreign bodies as compared with established methodologies [26].

Similarly, the ability to use TNE for management of pharyngeal and laryngeal foreign bodies was described by Sato and Nakashima at the School of Medicine, Kurume Japan. Seventeen patients, aged between eight and 89 years with complained of pharyngeal and laryngeal foreign bodies were included. They found that five foreign bodies located at the medial to the posterior portions of the lateral wall of the oropharynx, seven foreign bodies located at the anterior wall of the oropharynx, and one foreign body located at the supraglottis can be extracted with videoendoscope without a hood at its tip through the nasal passage (pernasal endoscopy) [27].

## 5. Safety

### 5.1. Complications of TNE

Studies have shown that TNE can be safely performed with topical anaesthesia in an office setting for diagnostic and therapeutic procedures [12–14, 16–18, 21, 22, 24, 25]. Aviv et al., McPartlin et al., Kumar and Amir, and LeBert et al. reported no complications associated with the use of TNE in their studies [12, 17, 18, 21, 25]. Epistaxis represents the most frequent minor complications of TNE (0.9% to 4.3%), but it was self-limited whereby bleeding was controlled with direct pressure [14, 16, 22]. Other minor complication encountered include vasovagal reaction that required no treatment (0.2% to 2.2%) [13, 14, 22]. Rees et al. in their study on transnasal balloon dilation of the oesophagus reported self-limited laryngospasm in one patient (1.8%) and intractable gagging in another patient (1.8%) [24]. In a study on placement of wireless pH capsule, Belafsky et al. reported laryngospasm in two patients (4.3%) [22]. One of the most feared complications of oesophagoscopy is oesophageal perforation. There was no reported oesophageal perforation or major complication associated with the use of TNE [12–14, 16–18, 22, 24, 25].

## 6. Patient Tolerance

Aviv et al. evaluated patients' tolerance by using a validated 10-point analog scale. Patients were asked to rate their level of tolerance to the nasal and oesophageal aspects of the procedure by assessing their anxiety, pain, and choking sensation or gagging. In all cases, the rating system was such that one represented no discomfort, well tolerated and ten represented severe discomfort, very poorly tolerated. The overall patient tolerance to TNE was rated as 2.0 (range, 1 to 4; standard deviation, 1.2). All patients indicate that they would repeat the TNE if requested by their physician [12]. Similarly, Price et al. in their study involving 116 patients also reported an average score of less than one for all types of discomfort on a visual analog scale of zero to ten [16]. Other studies have concluded that TNE was well tolerated by patients with local anaesthesia alone [13, 14, 17, 18, 21]. 

## 7. Cost/Cost-Effectiveness/Economic Evaluation 

No robust cost-effectiveness analysis (CEA) or cost utility analysis (CUA) regarding the economic value of TNE is available. However, two full text articles on economic evaluation related to the use of TNE were included in this report. As already been emphasized, sedation is not required for TNE. 

**Figure 2:**
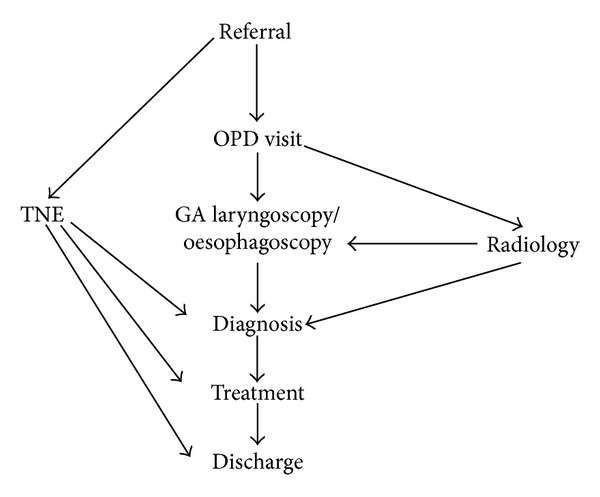
Patient pathway with and without TNE when investigated for a swallowing problem (dysphagia or globus).

Price et al. from UK evaluated the economic impact of using TNE (TNFLO) as the result of the shift from investigation and treatment in the operating theatre, to a procedure room-based practice under local anaesthesia. They demonstrated improved efficiency in management of certain patients such as patients investigated with swallowing problem (dysphagia or globus) with fewer steps involved in the pathway. By using TNE, referral for radiology investigations and laryngoscopy or oesophagoscopy under general anaesthesia can be reduced as shown in Figure 2.

This translates to monetary saving in terms of reduction in the reliance of radiological investigations (barium swallow ± *£*150), reduced follow-up clinic appointments (±*£*80), and a reduction in the cost of inpatient investigation with reduced admissions and use of in-patient theatres (general anaesthetic oesophagoscopy ± *£*450). Resource saving applies not only to the hospital, but also to the patient [16]. 

The cost implications of employing TNE as standard care for patients with globus pharyngeus and mild dysphagia were also analysed by McPartlin et al. from Charing Cross Hospital, London, UK. In their department, an average of 84 barium swallow investigations were requested annually for globus pharyngeus and mild dysphagia at the cost of *£*240 for investigation and *£*40 for a follow-up appointment (source: Trust's Finance Department). This brings the cost of the ‘postinitial consultation' investigations to *£*23,520 per annum. On the other hand, the capital cost of purchasing nasooesophagoscope was around *£*20,000, with disposable sheaths costing *£*50 each. Using these assumptions, the capital cost of purchasing a nasooesophagoscopoe will be fully met after 86 investigations, which was in their institution translates to just over one year of use. After that, the technology leads to savings of *£*230 (82%) per patient investigated for symptoms of upper aerodigestive tract pathology [17]. 

## 8. Other Considerations

### 8.1. Organizational

TNE is a new technology that allows the otolaryngologist to examine the upper aerodigestive tract from the nasal vestibule to the gastric cardia in the out patients department with topical local anaesthesia and without the need of sedation. An assessment of ease of use of equipment demonstrated that 43.7% (7/16) were rated as very easy to use and 56.3% (9/16) were rated as easy to use by the examiner. None were rated as difficult or very difficult to use [17]. Aviv et al. evaluated the ease of nasal insertion and oesophageal insertion using a validated 10-point analog scale. In all cases, the rating system was such that one represented extremely easy and ten represented extremely difficult. For the ease of nasal insertion, the mean rating was 1.3; range, 1 to 2, SD, 0.5 and for the ease of oesophageal insertion the mean rating was 2.9; range, 1 to 5; SD, 1.1, *P* < 0.001 [12]. In a study using office-based TNE to guide placement of the secondary TEP in 39 patients, technical difficulty in performance of the puncture was encountered in seven patients (17.9%) due to scar formation, nasopharyngeal stenosis, cervical oesophageal stenosis, and an aberrant course of the cervical oesophagus that was difficult to cannulate [25].

Price et al. found that TNE (TNFLO) takes an average of ten minutes to perform. The duration was slightly longer when therapeutic procedures were included (vocal cord medialization with collagen takes around 20 minutes). All patients treated in the outpatients were discharged within 2 hours. Using TNE (TNFLO), the authors have been able to discharge the majority of patients with globus (89.1%) and 47.8% of those with dysphagia after the initial visit to the department [16]. In a study for detection of metachronous oesophageal carcinoma in patients with HNSCC, Su et al. reported that the entire transnasal oesophagoscopy procedure time, including evaluation of upper aerodigestive tract and multiple biopsies of suspicious lesions, ranged from 10 to 40 minutes (median, 15 minutes) [21]. Bennett et al. reported an overall procedure time of less than 20 minutes, while recovery and discharge were possible one hour later in their study on management of patients with foreign bodies in the pharynx and oesophagus [26]. 

Two studies reported a change of practice with the introduction of TNE. Belafsky et al. reported that, in their department, TNE has replaced barium swallow as a screening examination of the oesophagus in patients with reflux, globus, and dysphagia [14]. Similarly, Price et al. highlighted that there has been a very substantial shift from investigation and treatment in the operating theatre to a procedure room-based practice under local anaesthesia [16].

Postma et al. and McPartlin et al. reported that TNE technique is an easy to learn procedure and the technique is quickly learned by operators familiar with the use of fibreoptic nasendoscopes [13, 17]. Falcone et al. conducted a study to determine the interobserver variability of findings reviewed by an otolaryngologist and a gastroenterologist. Fifty patients with throat symptoms presenting to the voice centre were asked prospectively to undergo TNE. The findings were videotaped and reviewed by an otolaryngologist and a gastroenterologist blinded to the patients presenting complaint. They found that 50% of patients were identified as having normal oesophageal findings by the gastroenterologist which was similar to the otolaryngologist findings (42%). The agreement was moderate, kappa score (*κ*) = 0.44 (CI, 0.19–0.68) with a percent agreement of 72%. The percent agreement (kappa scores) for various pathological findings was as follows: Barrett oesophagus 86% (*κ* = 0.45); oesophagitis, 88% (*κ* = 0.43); hiatal hernia, 76% (*κ* = 0.39); oesophageal stricture, 96% (*κ* = 0.73); patulous gastroesophageal junction, 98% (*κ* = 0.73); and oesophageal diverticulum, 100% (*κ* = 1.0) [28].

## 9. Discussion

There was no systematic review or HTA report retrieved. Cross-sectional studies revealed that TNE is well tolerated and can be safely performed in an office setting without the need for sedation. Among the thousands of TNE cases performed there was no reported oesophageal perforation or major complications. Minor complications were also uncommon. Because of its very nature, unsedated TNE eliminates all sedation-related events such as cardiopulmonary unplanned events secondary to conscious sedation [29]. 

The role of TNE continues to evolve in both diagnostic and therapeutic, particularly because of a high yield of pathology found on unsedated TNE examinations performed in an otolaryngology practice, with rates of pathological findings approaching 50 percent [13–15]. The main utility of office-based TNE is in the evaluation of patients with dysphagia or globus pharyngeus or reflux symptoms [12–18]. The review found that TNE may be used to perform a wide variety of procedures such as biopsies, placement of wireless pH capsule, transnasal balloon dilation of the oesophagus, secondary tracheoesophageal puncture, and management of foreign bodies with high success rate [13, 14, 16, 22–27]. The success rate of transnasal placement of wireless pH capsule was 85% compared with 89% via transoral [30]. The accuracy of biopsies taken using TNE matches those taken at standard panendoscopy [20]. Similarly, studies that compared unsedated transnasal EGD with sedated transoral EGD have shown no difference between the two techniques with respect to patient safety, feasibility, and tolerance [31–33].

Evidence showed that TNE can be performed within less than 20 minutes, while recovery and discharge were possible within one to two hours [16, 26]. In contrast, for rigid oesophagoscopy, patients need to be admitted for a few days [31]. The introduction of TNE has led to a change in practice such as TNE replacing barium swallow as screening examination in patients with reflux, globus pharyngeus, or dysphagia and also a substantial shift from investigation and treatment in operating room to procedure room-based practice under local anaesthesia [14, 16]. With this change, theatre resources can be more suitably utilized for procedures requiring a theatre environment. This may lead to potential direct cost saving compared with conventional oesophagoscopy [16, 17]. The increased direct costs of conventional oesophagoscopy, include longer procedure time, operation theatre, recovery room, recovery time, costs associated with investigations prior to general anaesthesia, medications, nursing, and monitoring. The resource savings apply not only to the hospital, but also to the patient in terms of days lost from work. 

Although TNE technique has been described as easy to learn procedure, the interpretation of the findings is challenging. Interpretations of TNE may vary within a speciality or between specialities [28].

TNE may be a substitute for conventional oesophagoscopy. However, there are certain instances in which one may prefer conventional oesophagoscopy. In cases in which it is expected that a significant time may be required to perform the procedure or in paediatric population, the surgeon may prefer the patient to be sedated [34].

## 10. Limitations

High-quality evidence is lacking. Most of the included studies were cross-sectional studies and retrospective in nature. There was only one study which provides evidence on diagnostic accuracy of TNE for patients with head and neck cancer. Although there was no restriction in language during the search, only English full text articles were included in the report. Although every effort has been made to retrieve full text articles, there were three abstracts for which the authors failed to retrieve full text.

## 11. Conclusion

Based on the review, there is evidence on the feasibility of TNE, particularly as a screening or diagnostic tool. More high-quality evidence is needed to assess its practicality for general use.
